# Genome-Wide Association Study Identifies *ALDH7A1* as a Novel Susceptibility Gene for Osteoporosis

**DOI:** 10.1371/journal.pgen.1000806

**Published:** 2010-01-08

**Authors:** Yan Guo, Li-Jun Tan, Shu-Feng Lei, Tie-Lin Yang, Xiang-Ding Chen, Feng Zhang, Yuan Chen, Feng Pan, Han Yan, Xiaogang Liu, Qing Tian, Zhi-Xin Zhang, Qi Zhou, Chuan Qiu, Shan-Shan Dong, Xiang-Hong Xu, Yan-Fang Guo, Xue-Zhen Zhu, Shan-Lin Liu, Xiang-Li Wang, Xi Li, Yi Luo, Li-Shu Zhang, Meng Li, Jin-Tang Wang, Ting Wen, Betty Drees, James Hamilton, Christopher J. Papasian, Robert R. Recker, Xiao-Ping Song, Jing Cheng, Hong-Wen Deng

**Affiliations:** 1 Key Laboratory of Biomedical Information Engineering of Ministry of Education, and Institute of Molecular Genetics, School of Life Science and Technology, Xi'an Jiaotong University, Xi'an, People's Republic of China; 2 Molecular and Statistical Genetics Lab, College of Life Sciences, Hunan Normal University, Changsha, People's Republic of China; 3 School of Medicine, University of Missouri Kansas City, Kansas City, Missouri, United States of America; 4 Department of Orthopaedics, the First Affiliated Hospital, School of Medicine, Xi'an Jiaotong University, Xi'an, People's Republic of China; 5 Department of Orthopaedics, Xiangya Hospital of Central South University, Changsha, Hunan, People's Republic of China; 6 Osteoporosis Research Center, Creighton University, Omaha, Nebraska, United States of America; 7 National Engineering Research Center for Beijing Biochip Technology, Beijing, People's Republic of China; 8 Center of Systematic Biomedical Research, Shanghai University of Science and Technology, Shanghai, People's Republic of China; University of Liège, Belgium

## Abstract

Osteoporosis is a major public health problem. It is mainly characterized by low bone mineral density (BMD) and/or low-trauma osteoporotic fractures (OF), both of which have strong genetic determination. The specific genes influencing these phenotypic traits, however, are largely unknown. Using the Affymetrix 500K array set, we performed a case-control genome-wide association study (GWAS) in 700 elderly Chinese Han subjects (350 with hip OF and 350 healthy matched controls). A follow-up replication study was conducted to validate our major GWAS findings in an independent Chinese sample containing 390 cases with hip OF and 516 controls. We found that a SNP, rs13182402 within the *ALDH7A1* gene on chromosome 5q31, was strongly associated with OF with evidence combined GWAS and replication studies (*P* = 2.08×10^−9^, odds ratio = 2.25). In order to explore the target risk factors and potential mechanism underlying hip OF risk, we further examined this candidate SNP's relevance to hip BMD both in Chinese and Caucasian populations involving 9,962 additional subjects. This SNP was confirmed as consistently associated with hip BMD even across ethnic boundaries, in both Chinese and Caucasians (combined *P* = 6.39×10^−6^), further attesting to its potential effect on osteoporosis. *ALDH7A1* degrades and detoxifies acetaldehyde, which inhibits osteoblast proliferation and results in decreased bone formation. Our findings may provide new insights into the pathogenesis of osteoporosis.

## Author Summary

Osteoporosis is a major health concern worldwide. It is a highly heritable disease characterized mainly by low bone mineral density (BMD) and/or osteoporotic fractures. However, the specific genetic variants determining risk for low BMD or OF are largely unknown. Here, taking advantage of recent technological advances in human genetics, we performed a genome-wide association study and follow-up validation studies to identify genetic variants for osteoporosis. By examining a total of 11,568 individuals from Chinese and Caucasian populations, we discovered a susceptibility gene, *ALDH7A1*, which is associated with hip osteoporotic fracture and BMD. *ALDH7A1* might inhibit osteoblast proliferation and decrease bone formation. Our finding opens a new avenue for exploring the pathophysiology of osteoporosis.

## Introduction

Osteoporosis, characterized primarily by low bone mineral density (BMD), is a major public health problem because it increases susceptibility to low-trauma osteoporotic fractures (OF). Hip fractures, which are the most common and severe form of OF, are associated with high morbidity and mortality, as well as tremendous health care expenditures [1]. Due to an aging population, the annual incidence of hip fractures worldwide is predicted to be ∼6.27 million by the year 2050, with an estimated cost of ∼$131.5 billion [1]. The ultimate goal of osteoporosis research is to reduce the incidence and prevalence of OF.

Genetic factors play an important role in susceptibility to osteoporosis. Both BMD and OF have high genetic determinations [2],[3],[4],[5]. BMD has been identified as the major risk factor for susceptibility to OF and is currently the predominant study phenotype for osteoporosis. Variations in BMD account for ∼50–70% of the variation in total bone strength [6] and risk of OF [7]. Additional risk factors, including those not readily quantifiable (e.g. bone microstructure [8] and cartilage organization [9]), also contribute to the risk of OF. Most of genetic studies of osteoporosis have focused primarily on the surrogate phenotype BMD, whereas little effort has been expended on the study of OF *per se* as a focal phenotype or on the relevance of genes associated with BMD on OF [3]. The major obstacle to this approach has been assembling a homogeneous sample with a homogenously defined OF type. Genetic factors associated with variations in BMD and risk of OF overlap, to some extent, but are not all identical [2]. The ultimate goal of osteoporosis research is to reduce the incidence and prevalence of OF. Therefore, it is useful to conduct genetic studies of OF *per se*, in conjunction with other intermediate phenotypes (e.g. BMD) that influence the risk of OF. This approach can be used to identify quantifiable measures for early prevention and intervention before the adverse clinical outcome, OF, actually occurs.

So far, several specific genes contributing to osteoporosis (i.e. those impacting BMD or risk of OF) have been identified, such as *ESR1* with OF risk, *COL1A1* and *VDR* with BMD and vertebral fracture risk, *OPG* and *LRP5* with BMD [10],[11],[12],[13],[14],[15]. However, the majority of genetic variants that influence osteoporosis remain unknown. With current high throughput SNP genotyping platforms and our knowledge about the distribution and correlation of SNPs in the human genome (e.g., haplotype structure), genome-wide association study (GWAS) has proven itself to be a feasible, powerful and effective approach for identifying novel genes associated with complex phenotypes. Four recent GWAS's [12],[13],[16],[17] have identified several specific genes for osteoporosis. In the current investigation, based on significant heritability of ∼50% for OF [2],[4], we utilized a GWAS to identify genetic variants underlying susceptibility to osteoporosis that are directly relevant to the risk of OF. Using the Affymetrix 500K array set, we successfully genotyped a study population of 700 elderly Chinese Han subjects consisting of 350 cases with homogeneous hip OF and 350 healthy matched controls. A follow-up replication study was performed in an independent Chinese sample consisting of 390 cases with hip OF and 516 controls. For SNPs that were identified for OF, we further examined their relationships with hip BMD in two ethnic groups (Chinese and Caucasians), involving additional 9,962 subjects, in order to determine whether the genetic basis for their contribution to the risk of OF might also be, at least partially, attributable to their effects on variation in BMD.

## Results

### GWAS Discovery Study

**Figure 1:**
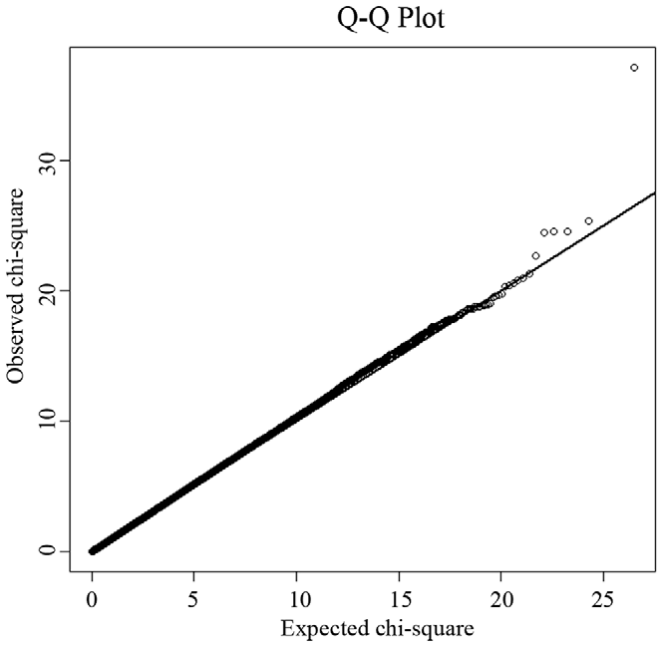
Quantile-quantile (QQ) plot of genome-wide allelic association results. Under the null hypothesis of no association at any locus, the points would be expected to follow the slope line. Deviations from the slope line correspond to loci that deviate from the null hypothesis.

The study design included an initial exploratory stage in a Chinese Han sample of moderate size and follow-up replication and validation studies with much larger sample sizes in independent Chinese Han and Caucasian samples. Table 1 details the basic characteristics of the respective samples. In the GWAS discovery stage, a total of 281,533 SNPs passed our quality control criteria for GWAS analyses. A quantile-quantile (QQ) plot is presented in Figure 1. The χ^2^ distributions for the association tests across the SNPs tested showed little evidence of overall systematic bias (genomic inflation factor λ = 1.02). The highest χ^2^ was consistent with the presence of true association. We further performed the principal component analysis implemented in EIGENSTRAT to guard against possible population stratification. The first two principal components were not associated with case status (*P* values>0.05), further indicating that it is very unlikely that positive associations in this study would be attributable to confounding due to population structure. The association analyses by EIGENSTRAT confirmed, qualitatively, our main results and consequently, the results of the EIGENSTRAT analyses are not detailed here.

**Table 1:** Characteristics of the study subjects.

	GWAS Sample		Chinese replication sample		Chinese BMD sample	US-MidWest samples		US-Framingham sample
	Case	Control	Case	Control		Unrelated	Related	
Number	350	350	390	516	2,955	1,725	2,329	2,953
Sex ratio (M/F)	124/226	173/177	112/278	188/328	1,437/1,518	868/857	843/1,486	1,274/1,679
Age (years)	69.4 (7.4)	69.5 (6.1)	68.1 (12.5)	68.2 (6.7)	33.1 (14.5)	55.0 (16.8)	49.0 (15.7)	63.1 (12.7)
Weight (kg)	59.2 (12.1)	59.6 (10.8)	56.8 (9.1)	62.6 (9.8)	58.6 (10.2)	80.9 (18.0)	77.4 (18.3)	76.5 (17.1)
Height (cm)	162.8 (8.3)	159.4 (9.2)	160.9 (7.4)	159.6 (8.5)	163.7 (7.9)	170.3 (9.8)	167.8 (10.2)	166.0 (9.9)

Abbreviations: M: male; F: female.

Data are shown as mean (standard deviation, SD).

Table 2 lists the most promising results from GWAS analyses. We identified five SNPs with *P* values<5×10^−6^ by allelic association analyses. After applying the Bonferroni correction for multiple testing, a single SNP, rs13182402, reached a genome-wide significance level (*P*<1.78×10^−7^). SNP rs13182402 achieved a *P* value of 8.53×10^−9^ in the allelic test (Bonferroni corrected *P* = 2.40×10^−3^). The odds ratio (OR) was 2.94 (95% confidence interval (CI): 2.02–4.30) for minor allele G. The frequency of the G allele was 0.162 in cases, and 0.061 in controls. When all covariates were considered simultaneously in a multivariate logistic regression model, this SNP remained a significant predictor of OF risk, independent of age, sex, height, and weight (*P* = 2.21×10^−8^).

**Table 2:** Summary of the GWAS and replication studies.

dbSNP	Chr	Position	Nearest Gene	Allele^a^	GWAS				Replication				Combined	
					MAF cases	MAF controls	*P* value^b^	OR (95% CI)	MAF cases	MAF controls	*P* value^b^	OR (95% CI)	*P* value	OR (95% CI)
rs13182402	5	125946047	*ALDH7A1*	G/A	0.162	0.061	8.53×10^−9^	2.94 (2.02∼4.30)	0.085	0.053	1.10×10^−2^	1.66 (1.12∼2.43)	2.08×10^−9^	2.25 (1.72∼2.94)
rs6039266	20	8714071	*PLCB1*	A/G	0.078	0.023	3.15×10^−6^	3.57 (2.03∼6.31)	-	-	-	-	-	-
rs6711417	2	170666928	*UBR3*	G/T	0.304	0.427	4.61×10^−6^	0.58 (0.46∼0.74)	0.356	0.370	0.521	0.94 (0.77∼1.52)	5.99×10^−4^	0.77 (0.66∼0.89)
rs16894980	8	122333958	*SNTB1*	A/G	0.123	0.055	4.71×10^−6^	2.43 (1.63∼3.61)	0.081	0.131	9.76×10^−3^	0.59 (0.39∼0.88)	0.094	1.27 (0.96∼1.67)
rs3212217	5	158687708	*IL12B*	C/G	0.384	0.415	4.85×10^−6^	0.88 (0.70∼1.10)	0.461	0.479	0.450	0.93 (0.77∼1.12)	0.212	0.91 (0.79∼1.05)
rs12100867	14	100582840	*DIO3*	G/A	0.251	0.359	1.19×10^−5^	0.59 (0.47∼0.75)	0.347	0.296	0.100	1.26 (0.96∼1.66)	0.023	0.81 (0.68∼0.97)
rs12590815	14	100573762	*DIO3*	C/T	0.196	0.292	3.07×10^−5^	0.59 (0.46∼0.76)	0.235	0.243	0.808	0.96 (0.71∼1.30)	8.29×10^−4^	0.72 (0.60∼0.87)
rs746219	20	57832814	*PHACTR3*	T/G	0.256	0.171	1.38×10^−5^	1.65 (1.28∼2.15)	0.231	0.226	0.804	1.03 (0.82∼1.29)	0.831	0.97 (0.82∼1.16)
rs6064822	20	57648532	*PHACTR3*	A/G	0.077	0.131	2.36×10^−5^	0.55 (0.38∼0.78)	0.086	0.079	0.612	1.09 (0.78∼1.52)	0.056	0.78 (0.62∼1.01)
rs1555364	20	57644265	*PHACTR3*	C/T	0.066	0.119	8.51×10^−5^	0.52 (0.36∼0.77)	0.096	0.071	0.178	1.38 (0.86∼2.23)	0.110	0.78 (0.58∼1.03)

Abbreviations: Chr: chromosome; MAF, minor allele frequency; OR, odds ratio; CI, confidence interval.

a The former allele represents the minor allele.

b All *P* values listed in Table 2 are two-sided.

### Assessment of Genome-Wide Findings

#### Replication in Chinese

Replication analyses were performed in an independent Chinese sample containing 390 cases with hip OF and 516 controls. Of the ten genotyped SNPs in the OF replication sample, two SNPs (rs13182402 and rs16894980) were nominally significant (Table 2). However, considering the effect direction, only SNP rs13182402 was successfully replicated (*P* = 1.10×10^−2^) and the effect was in the same direction as in the initial GWAS sample (OR: 1.66 for allele G, 95% CI: 1.12∼2.43). Combining the GWAS discovery and replication samples by meta-analyses, rs13182402 achieved a *P* value of 2.08×10^−9^ with an estimated OR of 2.25 (95% CI: 1.72∼2.94).

#### BMD validation in Chinese and Caucasians

To further explore the relationship between rs13182402 and osteoporosis risk, we performed association analyses with hip BMD in Chinese and Caucasian samples (Table 3). In the Chinese BMD sample, rs13182402 was associated with reduced hip BMD values (*P* = 2.35×10^−2^) and the effect size (β) was estimated to be ∼0.04 for each copy of the minor allele. This was consistent with its association with an increased risk of hip OF. The contribution of rs13182402 to BMD variation was estimated to be ∼0.68%.

**Table 3:** BMD validation association results for rs13182402.

Sample	Number	Alleles^a^	MAF	*P* value^b^	β^c^
Chinese BMD sample	2,955	G/A	0.062	2.35×10^−2^	−0.040
US-MidWest-unrelated sample	1,725	G/A	0.098	1.92×10^−2^	−0.043
US-MidWest-related sample	2,329	G/A	0.110	1.60×10^−3^	-
US-Framingham sample	2,953	G/A	0.084	3.38×10^−2^	-
combined samples	9,962			6.39×10^−6^	-

Abbreviations: MAF, minor allele frequency.

a The former allele represents the minor allele.

b *P* values were one tailed.

c The estimation for SNP effect size was performed under additive model.

d The *P* value from each sample set was combined based on the Stouffer method to quantify the overall association significance.

Statistical significance of rs13182402 was consistently achieved in the US-MidWest Caucasian samples (unrelated sample: *P* = 1.92×10^−2^; related sample: *P* = 1.60×10^−3^), and the effect is in the same direction as in the Chinese BMD sample. The β was estimated to be 0.043 for each copy of the minor allele in the unrelated sample. The contribution of this SNP to BMD variation in the unrelated sample was estimated to be ∼0.75%.

We further examined the association signal in the US-Framingham Caucasian sample. SNP rs13182402 was consistently significantly associated with hip BMD in the US-Framingham sample (*P* = 3.38×10^−2^).

We also examined the associations between rs13182402 and spine BMD in the Chinese and Caucasian BMD samples. However, no significant results were found (data not shown), which might be due to the heterogeneity of BMD across different skeletal sites [18].

Finally, using meta-analysis, we combined all of the BMD validation results (one Chinese sample and three Caucasian samples) to yield a commonly used probability measure. The statistical significance for rs13182402 was significantly improved (*P* = 6.39×10^−6^). These findings, combined with the results of our GWAS studies, lend strong support for the conclusion that rs13182402 is associated with low hip BMD and increased risk for OF.

### Fine Mapping for Gene Identification

**Figure 2:**
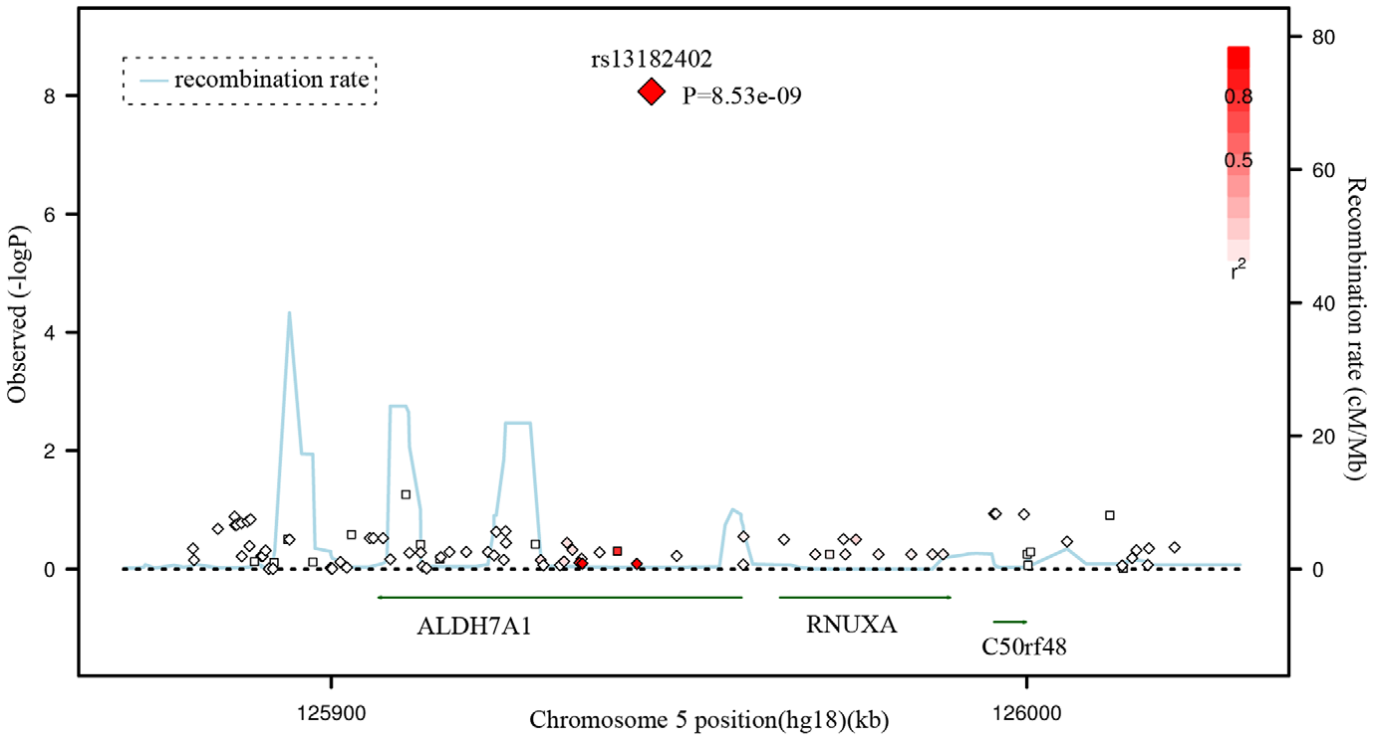
Regional Association Plot for rs13182402 on chromosome 5 in the GWAS stage. The color scheme of a white-to-red gradient reflects lower to higher LD values (*r^2^*). The scatter graph indicates the negative logarithm of *P*-value for each SNP.

Given the significant evidence for rs13182402, we imputed the genotypes of SNPs located surrounding this SNP based on our GWAS data and Asian HapMap data, and presented a regional association plot in Figure 2. The most significantly associated SNP, rs13182402 (GWAS: *P* = 8.53×10^−9^), is located 394 bp downstream from exon 5 of the *ALDH7A1* gene (aldehyde dehydrogenase 7 family, member A1) on chromosome 5q31. According to the FASTSNP program (http://fastsnp.ibms.sinica.edu.tw), a change of “A→G” at rs13182402 may lead to removal of binding sites for transcription factors *RORalp* and *CdxA*.

## Discussion

In this study, we first performed a GWAS and follow-up replication on OF and identified a novel susceptibility gene (*ALDH7A1*) that significantly impacts the risk for OF *per se*. Next, we examined this gene's relationship with hip BMD both in Chinese and Caucasian populations, and this gene was consistently associated with hip BMD even across ethnic boundaries. The effect size on BMD was modest and lower than the effect size on OF risk. One interpretation of this differential effect would be that BMD is not the only risk factor for OF; other risk factors also contribute to the risk of OF. It is consistent with and supports our statement in the introduction. It might also be caused by the differences in power between the relatively small hip OF samples compared to the large BMD samples. In addition, because we didn't have BMD measurements for the hip OF cases, we couldn't adjust the OR for BMD to see if the risk would be attenuated by the adjustment. However, regardless of this differential effect, the significant association results we identified both for BMD and OF risk strongly support the potential contribution of *ALDH7A1* to the pathogenesis of osteoporosis.

The *ALDH7A1* gene encodes an enzyme of the acetaldehyde dehydrogenase superfamily, which degrades and detoxifies acetaldehyde generated by alcohol metabolism. Acetaldehyde has been shown to inhibit osteoblast proliferation and to decrease bone formation [19]. In addition, previous studies have identified that polymorphisms of the *ALDH2* gene, another member of the acetaldehyde dehydrogenase family, are significantly associated with osteoporosis [20]. Our findings, combined with the above lines of evidence, suggest that *ALDH7A1* might be a novel and potential candidate gene contributing to the risk of osteoporosis.

Using the genotyped and imputed genotypes in our GWAS sample of 700 Chinese, we examined the associations between hip OF and the key SNPs identified in previous GWAS on osteoporosis [12],[13],[16]. Table 4 summarizes the major results. Only two SNPs in *RANKL* were confirmed to be associated with hip OF in our sample, including rs9594759 (*P* = 0.020) and rs9594738 (*P* = 0.045). The data provided may serve as a reference for other investigators searching for replication for their GWAS results.

**Table 4:** Comparison of the previous GWAS for BMD and the current GWAS.

SNP	Associated gene	Cytoband	Current GWAS *P* value	Published GWAS *P* value^a^	Reference
rs9479055	*ESR1*	6q25	0.936	7.0×10^−4^(hipBMD^b^)	[13]
rs1038304	*ESR1*	6q25	0.634	1.8×10^−5^(hipBMD)	[13]
rs6929137	*ESR1*	6q25	0.970	1.4×10^−5^(hipBMD)	[13]
rs1999805	*ESR1*	6q25	0.849	0.002(hipBMD)	[13]
rs2504063	*ESR1*	6q25	0.141	5.7×10^−8^(SPBMD^c^)	[13]
rs851982	*ESR1*	6q25	0.192	1.6×10^−5^ (hipBMD)	[13]
rs4870044	*ESR1*	6q25	0.751	9.9×10^−5^ (hipBMD)	[13]
rs3736228	*LRP5*	11q13	0.684	1.9×10^−5^ (SPBMD)	[12]
rs2010281	*MARK3*	14q32	0.331	7.4×10^−5^ (hipBMD)	[16]
rs4355801	*OPG*	8q24	0.481	7.9×10^−4^(SPBMD)	[12]
rs9594759	*RANKL*	13q14	0.020	4.5×10^−4^ (hipBMD)	[13]
rs9594738	*RANKL*	13q14	0.045	2.7×10^−4^(hipBMD)	[13]
rs3018362	*RANK*	18q21	0.185	3.5×10^−5^(hipBMD)	[16]
rs1513670	*SOST*	17q21	0.385	6.0×10^−5^(hipBMD)	[16]
rs7220711	*SOST*	17q21	0.930	1.4×10^−4^ (hipBMD)	[16]
rs1107748	*SOST*	17q21	0.930	7.2×10^−5^(hipBMD)	[16]

a *P* value reported here was the original *P* value in the discovery sample in each GWAS.

b hipBMD is the combined BMD at the femoral neck, trochanter and intertrochanter region.

c SPBMD: Spine BMD.

An apparent advantage of this study is that our GWAS sample came from a homogenous population with well defined homogeneous phenotype. The genomic control factor was quite close to 1.0 (λ = 1.02) (expected under no population stratification) and, analyses by EIGENSTRAT showed qualitatively supportive results. Thus, our association results are unlikely to be plagued by spurious associations due to population stratification. In particular, since the significant associations with BMD are shown in both Caucasian and Chinese samples, the results are even less likely to be due to population stratification/admixture.

A potential limitation of our study is the relatively small size of the GWAS sample and the replication sample, which might lead to over estimation of the effect size for the significant SNPs identified. However, hip fractures are the most severe OFs followed by high mortality rates, making subjects recruitment difficult. It took us several years to accumulate such a homogeneous hip OF sample. This study represents the best we can do under current conditions to identify genes for OF. Meanwhile, we are keeping the recruitment of hip OF subjects. As a future direction, a new GWAS needs to be implemented on a larger sample to identify more comprehensively novel genes for OF. In addition, since genetic and environmental backgrounds vary for different populations, replication across a wide range of populations is necessary to determine the generality of our findings to the broader population, or to specific ethnic groups or populations [21].

In summary, using data from over 11,500 individuals, we have identified and validated *ALDH7A1* as a novel susceptibility candidate gene for osteoporosis. Further studies are warranted to explore the generality of our findings for *ALDH7A1* identified by GWAS to other populations, and to determine the mechanisms by which this gene and its products contribute to the pathogenesis of osteoporosis.

## Materials and Methods

### Ethics Statement

The study was approved by the local institutional review boards or the office of research administration of all participating institutions. After signing an informed consent, all subjects received assistance completing a structured questionnaire including anthropometric variables, lifestyles, and medical history.

### Study Samples

The study was initially performed with a GWAS discovery stage for SNPs of potential significance for OF in a Chinese Han, case-control sample. The significance of the SNPs identified in the discovery stage was subsequently confirmed through replication study in another independent Chinese case-control sample. For SNPs identified for OF, we further examined their relationships with hip BMD within/across ethnic groups in a Chinese unrelated BMD sample and three independent Caucasian samples. Table 1 details the basic characteristics of the respective samples, with additional descriptions below.

### GWAS Discovery Sample

The sample for the initial GWAS consisted of 350 patients with osteoporotic (low trauma) hip fractures (including 124 males and 226 females) and 350 elderly controls (including 173 males and 177 females). Since fractures at different skeletal sites may have different underlying pathological mechanisms, we focused exclusively on hip fractures in order to minimize potential clinical and genetic heterogeneity of the study phenotype. All the subjects were unrelated northern Chinese Han adults living in the city of Xi'an and its neighboring areas. Affected individuals with low trauma hip fractures were recruited from the affiliated hospitals and their associated clinics of Xi'an Jiaotong University. Inclusion criteria for cases were (i) onset age of hip OF>55 years, to make sure all female subjects were postmenopausal, and the onset of OF was largely due to decreased BMD; (ii) age<80 years to minimize the effect due to age, since previous studies showed that approximately half of females aged 80 years or older have fractures [22]; (iii) fractures occurred with minimal or no trauma, usually due to falls from standing height or less; (iv) the fracture sites were at the femoral neck or inter-trochanter regions; (v) hip fracture was identified/confirmed through diagnosis of orthopedic surgeons/radiologists according to radiological reports and x-rays. Patients with pathological fractures and high-impact fractures (such as due to motor vehicle accidents) were excluded. Patients with chronic diseases before the onset of HF were also excluded.

Healthy control subjects were enrolled by use of local advertisements. They were geography- and age-matched to the cases. Inclusion/exclusion criteria for controls were: (i) age at exam must be >55 years, without any fracture histories (all female controls were postmenopausal); (ii) subjects with chronic diseases and conditions that might potentially affect bone mass, structure, or metabolism were excluded. Diseases/conditions resulting in exclusion included chronic disorders involving vital organs (heart, lung, liver, kidney, brain), serious metabolic diseases (diabetes, hypo- and hyper-parathyroidism, hyperthyroidism, etc.), other skeletal diseases (Paget's disease, osteogenesis imperfecta, rheumatoid arthritis, etc.), chronic use of drugs affecting bone metabolism (e.g., hormone replacement therapy, corticosteroid therapy, anti-convulsant drugs), and malnutrition conditions (such as chronic diarrhea, chronic ulcerative colitis); (iii) subjects taking anti-bone-resorptive or bone anabolic agents/drugs, such as bisphosphonates were excluded.

### Chinese Replication Sample

For replication of our GWAS findings for hip OF, we used an independent Chinese sample containing 906 unrelated Han subjects (390 cases with hip OF and 516 controls). All subjects were drawn from the same geographic area as the above GWAS discovery sample, and the sample inclusion and exclusion criteria for cases and controls were the same as those adopted in the recruitment of the above GWAS sample.

For SNPs that were identified for OF, we further performed validation analyses to evaluate their relevance with hip BMD (with targeted experimental genotyping of candidate SNPs discovered in the initial GWAS) in two ethnic groups, including a Chinese sample and two US Mid-West Caucasian samples. We finally performed *in silico* validation to compare the association signals of our most promising GWAS results with those achieved in the Framingham Heart Study (FHS) [23].

### Chinese BMD Sample

The Chinese BMD sample contained 2,955 unrelated ethnic Han adults. This sample came from Changsha, Hunan province, which is more than 1,000 km from Xi'an where the sample for the GWAS was recruited. The subjects were randomly selected from an established and expanding database with BMD measurements. The exclusion criteria were the same as those adopted in the recruitment of healthy control subjects in the GWAS sample, and have been detailed in our earlier publication [2].

### US–MidWest Caucasian BMD Samples

The US-MidWest BMD samples with a total of 4,054 subjects consisted of two independent sample sets, including one sample of unrelated subjects and the other sample of nuclear families, which were all US Caucasians of Northern European origin living in Omaha, Nebraska, and its surrounding regions in Midwestern USA. They were normal healthy subjects defined by the same exclusion criteria as above in Chinese samples. The unrelated sample contained 1,725 subjects. The related sample contained 2,329 subjects from 593 nuclear families.

All hip BMD measurements for the above BMD samples were obtained with dual-energy X-ray absorptiometry using the same type of machine (Hologic 4500) under the same protocol defined by the manufacturer (Hologic Inc., Bedford, MA, USA). The machines were calibrated daily. The coefficients of variation (CV) of the hip BMD measurements were 1.33% for Chinese and 1.40% for Caucasians, respectively.

### US-Framingham BMD Sample

The US-Framingham BMD sample is from the Framingham Osteoporosis Study, an ancillary study of the Framingham SNP Health Association Resource (SHARe) data sets [23]. Details and descriptions about the Framingham Osteoporosis Study have been previously reported [24]. Both genotype and phenotype data were downloaded from dbGaP database (http://www.ncbi.nlm.nih.gov/sites/entrez?db=gap). Data download and usage was authorized by SHARe data access committee (phs000007.v3.p2, phs000078.v3.p2). We have the data on 2,953 phenotyped Caucasian subjects, 448 from the Original cohort (160 men and 288 women) and 2,505 from the Offspring cohort (1,114 men and 1,391 women). The Original Cohort participants had BMD measures by dual x-ray absorptiometry machine (Lunar DPX-L) at the hip performed at exam 24. The Offspring Cohort participants were scanned with the same machine at exam 6/7. As reported before [24], the CV was 1.7% for femoral neck.

### Genotyping and Quality Control

Genomic DNA was extracted from peripheral blood leukocytes using standard protocols. The genome-wide scan was performed using the Affymetrix Human Mapping 500K array set (Affymetrix, Santa Clara, CA, USA) according to the Affymetrix protocol. Data management and analyses were conducted using the Affymetrix GeneChip Operating System. Genotyping calls were determined from the fluorescent intensities using the DM algorithm with a 0.33 *P*-value setting [25] as well as the B-RLMM algorithm [26].

Quality control procedures were as follows. First, only samples with a minimum call rate of 95% were included. Due to efforts of repeat experiments, all samples (*n* = 700) met this criteria and the final mean BRLMM call rate reached a high level of 99.02%. Second, out of the initial full-set of 500,568 SNPs, we discarded: 1) SNPs with a call rate <90% in the total sample (*n* = 54,845); 2) those deviating from Hardy-Weinberg equilibrium (HWE) in controls (*P*<0.001, *n* = 22,002); 3) those having a minor allele frequency (MAF)<0.05 in the total sample (*n* = 142,188). Therefore, 281,533 SNPs were available for subsequent analyses.

Based on the initial GWAS results, we selected the 10 most promising SNPs for subsequent genotyping in the Chinese hip OF replication sample based on the following inclusion criteria: (i) *P* values≤5×10^−6^ in the GWAS allelic association analyses (5 SNPs); (ii) *P* values between 5×10^−6^ and 5×10^−5^, with neighboring SNPs having *P* values≤10^−4^ showing a consistent trend of association (5 SNPs). Genotypes were obtained using MALDI-TOF mass spectrometry on a Sequenom system (Sequenom, Inc., San Diego, CA) with iPLEX assay [27]. Primers were designed using MassARRAY Assay Design 3.1 software. Genotyping quality control procedures leading to SNP exclusion were call rate <90%, MAF<0.05 in the total sample and *P*<0.001 for deviations from HWE in controls. The average call rate was 98.7% for the Sequenom system and the corresponding consistency of genotyping (replication or concordance rates), as obtained by duplication samples, was 99.8%. Nine of the ten genotyped SNPs were qualified for subsequent association analyses.

The Chinese BMD sample was also genotyped using the Sequenom system, which was the same as that used for the OF replication sample. Genotyping of the two US-MidWest samples was performed by a service company KBioscience (Herts, UK) using a technology of competitive allele specific PCR (KASPar), which is detailed at the website (www.kbioscience.co.uk). The US-Framingham sample was genotyped using approximately 550,000 SNPs (Affymetrix 500K mapping array plus Affymetrix 50K supplemental array).

### Statistical Analyses

For the GWAS analyses for OF risk, single-marker allelic association analysis was performed by comparing SNP allele counts among cases and controls with a χ^2^ test. ORs with the corresponding 95% CIs were also computed. For the interesting SNPs identified by allelic tests, we also used a multivariate logistic regression model to examine associations with OF risk, taking into account potential covariates such as age, sex, height, and weight. For the sex chromosome analyses, the affymetrix platform does not assay the Y chromosome. The X chromosome needs to be treated differently from the autosomes since males have only one copy of the X chromosome. As most loci on the X chromosome are subject to X chromosome inactivation, it is reasonable to treat males as if they were homozygous females, and then the assumption was the same as tests for autosomes. All association statistical analyses were carried out using HelixTree 5.3.1 software (Golden Helix, Bozeman, MT, USA). We adjusted for multiple testing by adopting the conservative Bonferroni correction. The genome-wide significance threshold was set at a *P* value of less than 1.78×10^−7^ (0.05/281,533 SNPs that passed our quality control check).

To correct for potential population stratification that may lead to spurious association results, we estimated the inflation factor (λ) for the GWAS sample using a method of genomic control [28]. λ was calculated as the median of the observed χ^2^ statistics divided by the median of the expected χ^2^ statistics for the genome-wide SNP set. This led to an λ of 1.02. Results presented in this study were based on adjusting χ^2^ statistics by dividing each of them by 1.02. The data were also analyzed by the principal component analyses implemented in EIGENSTRAT [29] for cross-checking the association results while controlling for admixture.

For OF replication analyses, the same allelic association analysis was performed by χ^2^ tests. For BMD validation analyses, significant parameters (*P*<0.05) such as age, sex, height and weight were used as covariates to adjust for the raw BMD values. For the unrelated samples, ANOVA was conducted to achieve the association tests. ANOVA is a model free test and more robust than assuming any genetic models. The independent variable was the genotype, which was divided into three levels corresponding to the three genotypes observed for each SNP (1,1; 1,2; 2,2). Since ANOVA can't give the effect size, we estimated the effect size of significant SNPs using the linear regression assuming the additive model in SAS (SAS Institute Inc., Cary, NC). For the related samples, we used the BMD residuals after adjustment to conduct family-based association tests under an additive model using FBAT program [30]. FBAT is a powerful approach to handle family sample, which tests for differences in probability of transmission of a genotype from parents to offspring based on phenotype. FBAT examines association within families, which is not affected by population stratification bias. To quantify the overall evidence of associations, we performed meta-analyses by using the Mantel-Haenszel method to calculate the *P* values and OR for the combined OF samples. For the BMD samples, we used a weighted Z-score method to calculate the combined *P* values. The individual Z-score (a standard normal deviate, the statistic associated with a *P* value) was weighted by the square root of the sample size of each sample. We added the individual weighted Z-score together and divided by the square root of the total sample size to obtain a combined Z-score and an associated combined *P* value (Stouffer method) [31]. If an individual result was nonsignificant and gave no other useful data for calculation of a Z-score, we set it as 0 to calculate the combined probability.

For the interested genomic regions, IMPUTE program [32] was utilized to impute the genotypes of all SNPs located in the regions based on Asian HapMap data. SNPTEST [32] was used to test for associations between the imputed SNPs and OF. SNAP was used to depict the regional association plot [33].
